# Systematic quantitative modeling of the natural history of Aicardi syndrome: A cross sectional study of 245 published cases

**DOI:** 10.1186/s13023-024-03375-8

**Published:** 2024-12-04

**Authors:** Oliver Y. Urban, Jan H. Driedger, Sven F. Garbade, Georg F. Hoffmann, Stefan Kölker, Markus Ries, Steffen Syrbe

**Affiliations:** 1 Division of Pediatric Epileptology, Center for Pediatric and Adolescent Medicine, Clinic 1, Medical Faculty of Heidelberg, Heidelberg University, Im Neuenheimer Feld 430, 69120 Heidelberg, Germany; 2 Division of Pediatric Neurology and Metabolic Medicine, Center for Pediatric and Adolescent Medicine, Clinic 1, Medical Faculty of Heidelberg, Heidelberg University, Heidelberg, Germany

**Keywords:** Aicardi syndrome, Natural history, Epileptic encephalopathy, Infantile spasms, Agenesis of corpus callosum, Chorioretinal lacunae

## Abstract

**Purpose:**

Aicardi syndrome is a rare epileptic encephalopathy, almost exclusively affecting girls. It was first described as a triad of infantile spasms, chorioretinal defects and agenesis of the corpus callosum. The etiology remains unknown and there is uncertainty on best practice therapy and outcome. We aimed at defining quantitative clinical endpoints that will inform future research and clinical trials.

**Methods:**

Quantitative natural history modeling of cases with Aicardi syndrome from published clinical reports. Main outcome measures were age at disease onset, survival and diagnostic delay. Phenotypic features of affected individuals as well as neuroradiological and ophthalmological features were descriptively stated. STROBE criteria were respected.

**Results:**

Two hundred forty-five cases were available for analysis. Median age at disease onset was 2.2 months. Median diagnostic delay was 1 month. Mortality was estimated with 6% at 1 and 17% at 5 years of age. 60% of children showed the classic clinical features, while 40% met the revised diagnostic criteria. We delineate possible predictors of disease severity and of seizure control.

**Conclusion:**

We provide natural history data including geographical localization of 245 published patients with Aicardi syndrome. Quantitative history modeling in rare epileptic encephalopathies will help to raise disease awareness and facilitate future clinical trials as one core element of quantitative systems pharmacology.

**Supplementary Information:**

The online version contains supplementary material available at 10.1186/s13023-024-03375-8.

## Introduction

Aicardi syndrome (AS, OMIM %304050) is a severe developmental and epileptic encephalopathy. It was defined as a triad of infantile spasms, agenesis of the corpus callosum (ACC) and chorioretinal lacunae (CRL), exclusively affecting girls, by Aicardi in 1965 [1, 2]. AS is a rare disease [3] with an estimated incidence in the United States of 1 per 105,000 live-births [4].

With an increasing number of reported patients and improved brain imaging, additional features and a large variability of the phenotype became apparent. The spectrum of neuroimaging findings in AS now includes neuronal migration and postmigrational disorders, ventriculomegaly, diverse cysts, asymmetry of the cerebral hemispheres, cerebellar anomalies, and papillomas of the choroid plexus. Malformations of cortical development (MCD), most frequently polymicrogyria and periventricular or subcortical heterotopias, midline or choroid plexus cysts and optic disc coloboma or optic nerve hypoplasia, have been reported as major features [5]. Other associated findings are skeletal anomalies, such as scoliosis, hemi- or butterfly vertebrae and missing or additional ribs. Ophthalmologic anomalies include colobomas of the optic disc or iris [6, 7], persistent fetal vasculature [6, 8, 9], microphtalmia [6], optic nerve dysplasia, retinal detachment, and nystagmus [6, 10].

Seizures usually manifest as infantile spasms with an onset in the first months of life [11]. Other seizure types are frequent [12, 13]. Severe intellectual disability and movement disorders are regularly seen [14–17]. Even with a growing number of reports, there is an uncertainty of the natural course of the disease and predictors of the severity of the disease are lacking.

The purpose of this study was to quantitate the natural history of the condition based on published cases from the literature, thereby facilitating better counseling of affected families and enabling targeted future research [18]. Quantitative natural history modeling based on published cases has not been applied in Aicardi syndrome. The present paper aims to close this gap. Primary hard clinical endpoints were age at onset, age at diagnosis and survival. Secondary endpoints were clinical characteristics of the population and possible predictors of clinical outcome.

## Methods

Quantitative natural history modeling based on published cases (thoroughly reviewed in Garbade et al. [18]) is a clinical-statistical technique to qualitatively define the natural history of a condition and can be used as one core element in the overall scientific context of quantitative systems pharmacology [19]. This study was planned, executed and reported in accordance with the STROBE (Strengthening the Reporting of Observational studies in Epidemiology) criteria.

We conducted a literature search on PubMed for the term ‘Aicardi syndrome’ excluding the term ‘Goutières’, hereby excluding the easily confounded, but entirely different disease ‘Aicardi-Goutières syndrome’. Identified publications were downloaded and manually reviewed for eligibility concerning sufficient clinical and/or genetic data. Qualifying reports were published between 1969 and 2019 and in total 245 patients out of 118 publications qualified for further analysis. Close of database was on 31 January 2019.

The following variables were extracted: year of publication, sex, origin, family history concerning siblings and parents, age at disease onset, age at diagnosis, last reported age, information whether the patient is deceased or alive, leading symptoms, neuroradiological and ophthalmological information, facial and physical features, mode and effects of treatment and possible complications. If the origin of a patient was not explicitly stated in the published report, we assumed the country of the corresponding author’s institutional affiliation to be the country of patient’s origin. Patients were considered alive at the time of the report if not explicitly stated otherwise.

Inclusion criteria were the revised diagnostic criteria proposed by Sutton et al. (Supplementary Table 1) defined by the presence of two classic features, i.e. agenesis of the corpus callosum, infantile spasms or CRL, as well as at least two major features of AS.

Seizure control and intellectual disability were acquired in semiquantitative terms, either by specific statements in the original reports, or when the same allowed for a reasonably well-founded interpretation.

### Statistical analysis

Descriptive statistics for continuous variables will be described by median and interquartile range (IQR). All neuroimaging features were dichotomized as being present or not, independent of their severity. Variables were illustrated by giving counts and percentages according to the respective sample size. Survival was defined as the difference in time between birth and time of death. Survival was estimated by using the Kaplan–Meier estimator. Log-rank test was used to compare subgroups in survival analysis. Degree of seizure control (good, moderate and insufficient) over age at first epileptic seizure was analyzed with proportional odds logistic regression (according to Agresti 2002 a ‘cumulative link model’ [20]). The impact of age at onset and sidedness of chorioretinal lacunae on survival was analyzed using unbiased recursive partitioning. Diagnostic delay was defined as the difference between age at onset and age at diagnosis. The world map was plotted using the R extension ggmap. The wordle figure was created using an open-source web-based tool (https://www.freewordcloudgenerator.com/generatewordcloud) [21]. Pearson’s Chi-squared tests were carried out for all neuroimaging features concerning their effect on seizure control and seizure freedom.

When data was missing in single subsets, patients were excluded for these specific subsequent data analyses. Respective sample sizes (N) were indicated for the corresponding analyses. Sensitivity analyses were not conducted. All analyses were conducted using R (http://www.r-project.org) or Microsoft Excel (https://www.microsoft.com/en-us/microsoft-365/excel). *P* ≤ 0.05 was considered statistically significant. Anonymized data not published within this article and the analysis plan and applied R-script will be made available by request from any qualified investigator.

### Ethical considerations

This study did not directly involve human subject research, because data were extracted from published papers in the medical literature. We confirm that we have read the Journal’s position on issues involved in ethical publication and affirm that this report is consistent with those guidelines.

## Results

**Fig. 1:**
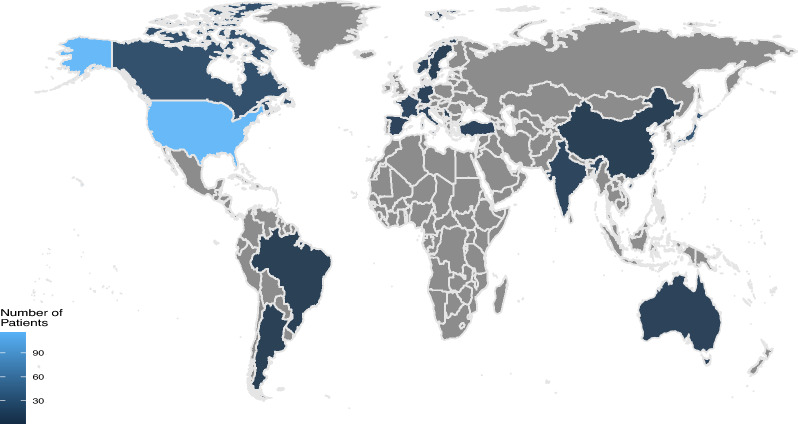
Geographical distribution pattern of patients with Aicardi syndrome. Gray scale indicates the number of identified affected individuals per country. Dark blue: 1 to 30 patients, medium blue: 30 to 90 patients, light blue: more than 90 patients. If the origin of a patient was not explicitly stated in the published report, we assumed the country of the corresponding author’s institutional affiliation to be the country of patient’s origin

We identified 245 individuals from 118 case descriptions or case series (published between 1965 and 2019) for further statistical analysis. The characteristics of the study cohort are depicted in Table 1. All included children presented either with the original triad or were diagnosed according to the revised diagnostic criteria. Almost all affected individuals were of female sex. Seven individuals presented a male phenotype but a chromosomal genotype of XX or 47, XXY. The median age was 3.0 years at last follow-up. The geographical origin of afflicted individuals is illustrated in Fig. 1.

**Table 1:** Characteristics of the study cohort of patients with Aicardi syndrome (N = 245)

Population characteristics	N	Percent (%)
Sex		
Female	238	97
Male	7	3
Diagnostic criteria		
Classic triad*	146	60
Revised	99	40
Alive at last follow up		
Yes	194	79
No	33	14
Unreported	18	7

Clinical characteristics of 245 patients with Aicardi syndrome. Almost all are of female sex and the majority shows the classic triad

*The classic triad of Aicardi syndrome consists of infantile spasms, ACC and CRL

### Confirmation of diagnosis

One hundred forty-six of 245 individuals (60%) had the classic triad of AS, 99 (40%) cases showed two classic and at least two other associated features enabling the diagnosis of AS. Most often diagnostic work-up was prompted by infantile spasms or developmental delay. Information on mode of diagnosis was stated in 191 cases, and MCDs were identified with MRI (136, 71%), CT-Scan (35, 18%), autopsy (12, 6%), cranial sonography (4, 2%), or pneumoencephalography (4, 2%).

### Age at disease onset

The median age at onset (first occurring symptoms) was 2.2 months, interquartile range (IQR) from 1.0 to 3.0 months (N = 166). The median age at onset of infantile spasms was equally 2.2 months, with an IQR of 1.6 months (N = 104).

### Diagnostic delay

**Fig. 2:**
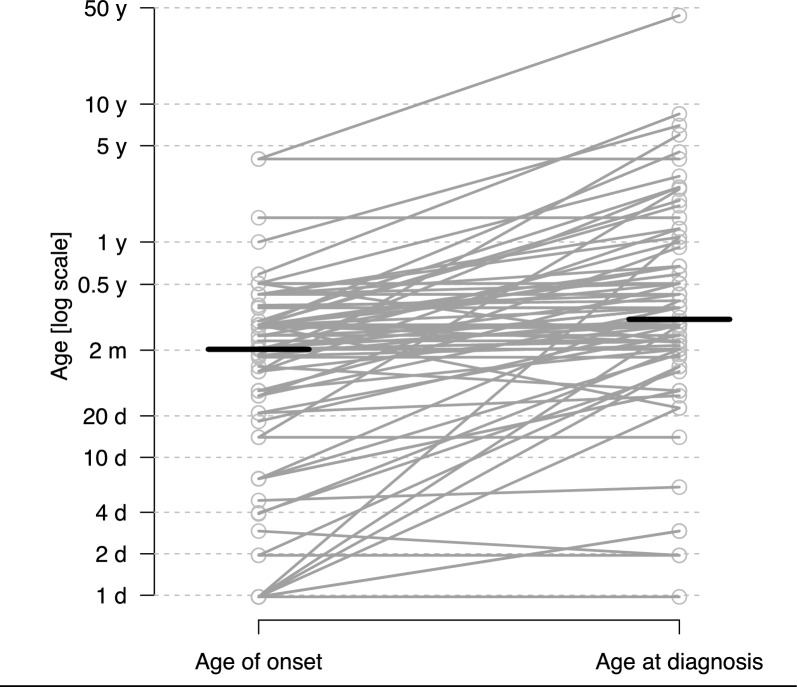
Age at onset (in months) of Aicardi syndrome and age at diagnosis. Data available for 86 patients. Horizontal lines indicate the medians. The slopes of connecting lines represent the delays between onset of the disease and times of diagnosis

Time to diagnosis was calculated from age at disease onset and age at diagnosis (N = 86). Median age at diagnosis was 3.3 months, IQR from 2.0 to 7.75 months with a median delay to diagnosis of 1.0 month, IQR from 0 to 4.2 months (Fig. 2).

### Survival estimations

**Fig. 3:**
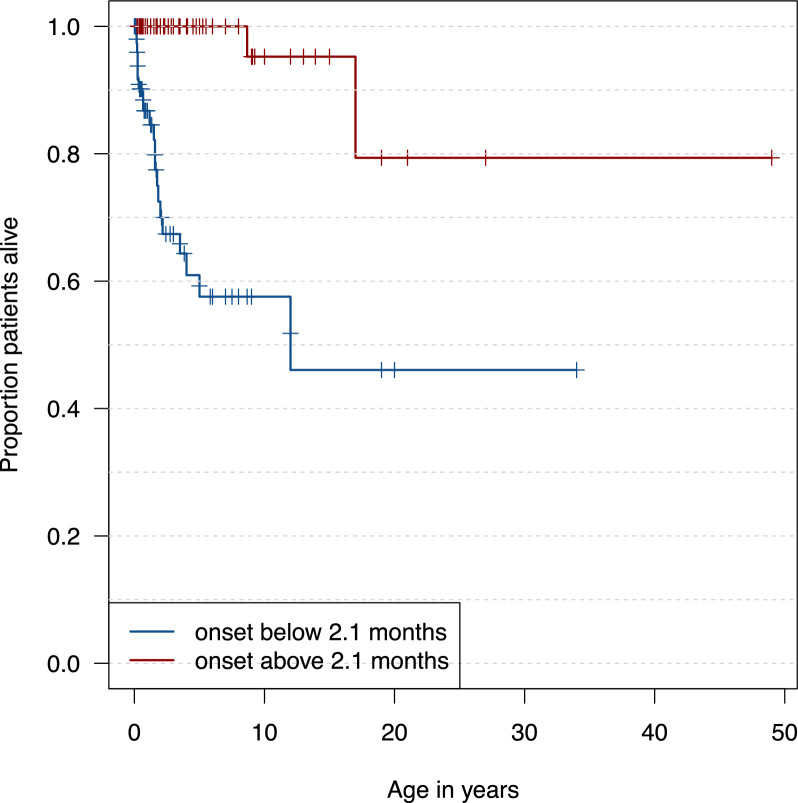
Estimated survival distributions for patients with Aicardi syndrome (N = 227) in relation to age at onset. Censored input is depicted by a vertical line. The red line delineates the estimated survival for age at onset below 2.1 months. The blue line delineates the estimates survival for age at onset above 2.1 months

Last reported status and age could be assessed in 227 individuals. In this population 194 children (85%) were still alive and 33 (15%) were deceased at time of reporting. Survival estimations using the Kaplan–Meier-method showed that 94% of patients were alive at 1 year of age and 83% of patients were alive at 5 years of age (Fig. 3, supp. table 7). Survival was significantly worse for disease onset before the age of 2.1 months (*p* = 0.01) (Fig. 3, eFigure 1).

### Clinical characteristics of children with Aicardi syndrome

#### Development

Data on intellectual functioning was available from 169 cases, with 163 (96%) cases displaying intellectual disability (ID). The level of ID was stated in 134 cases, with severe, moderate and mild ID occurring in 100 (75%), 16 (12%) and 18 (13%) individuals respectively. Observations on verbal functioning were available in 87 cases. There was no communication possible or restriction to non-verbal communication in 33 (38%) and 28 (32%) cases respectively. Expression by simple sounds was possible in 11 (13%) children. Words or full sentences could be formed by 11 (13%) and 4 (5%) children respectively. Mode of locomotion was described in 114 children. 62 (54%) of whom were unable to move at all. The Capability to roll around or walk with assistance was present in 12 and 13 children respectively. 27 (24%) children were able to walk unassisted. Abnormal muscle tone was reported in 85 (35%) cases of the entire cohort.

#### Epilepsy and semiology

In 239 (98%) cases epilepsy was reported with information on semiology available for 221 (90%) children. The most common seizure types at diagnosis were infantile spasms in 190 (86%, N = 221) and focal seizures in 38 (17%) cases. Other reported seizures at diagnosis were generalized tonic–clonic (17, 8%), generalized tonic (13, 6%), generalized clonic (7, 3%), generalized atonic (4, 2%), absences (7, 3%), myoclonic (11, 5%) and other not specified (4, 2%) seizures. Three or more different semiologies were reported in 8 (4%) infants. Seizures that were reported in addition to the dominant semiology at time of diagnosis were infantile spasms (36%), focal (28%), generalized tonic–clonic (26%) or tonic (24%) seizures. Supp. Table 2 shows the complete spectrum of seizure types at diagnosis and at last follow-up.

#### EEG findings

Electroencephalography (EEG) was pathological in 128 (99%, N = 130) cases at time of diagnosis. Asymmetry of both hemispheres in 43% and hypsarrhythmia in 41% were the most common findings. Other findings included focal and generalized epilepsy-typical potentials (ETP) in 37% and a burst-suppression pattern in 16% of cases. At last follow-up ETPs and asynchronous activity were most common (57% and 41% respectively, N = 70). Persisting hypsarrhythmia at last follow-up was reported in 29% of children with a median age of 13.5 months up to a maximum age of 36 months [22] in single children (IQR 48.8 months).

#### Neuroimaging and ophthalmological findings

An abnormal corpus callosum was present in 240 (98%) children. It was described as complete ACC in 163 (67%), as incomplete in 42 (17%) and as hypoplastic in 9 (4%) cases. It was not further specified in 26 (11%) children. Polymicrogyria was found in 52 (21%) cases. It was bilateral in 30 (12%) cases and did affect only the right or left hemisphere in 4 and 5 (2%) cases respectively. Schizencephaly was found in 5 (2%) cases. Intracranial cysts were found in 86 (35%) children. They were described as interhemispheric, arachnoid, porencephalic, choroid plexus and interventricular cysts in 39 (16%), 21 (9%), 11 (5%), 9 (4%) and 6 (2%) cases respectively. Subcortical heterotopias were present in 43 (18%) and periventricular nodular heterotopias in 18 (7%) cases. Unspecified cortical dysplasia was reported in 21 (9%) children. Choroid plexus papilloma was present in 10 (4%) individuals.

**Fig. 4:**
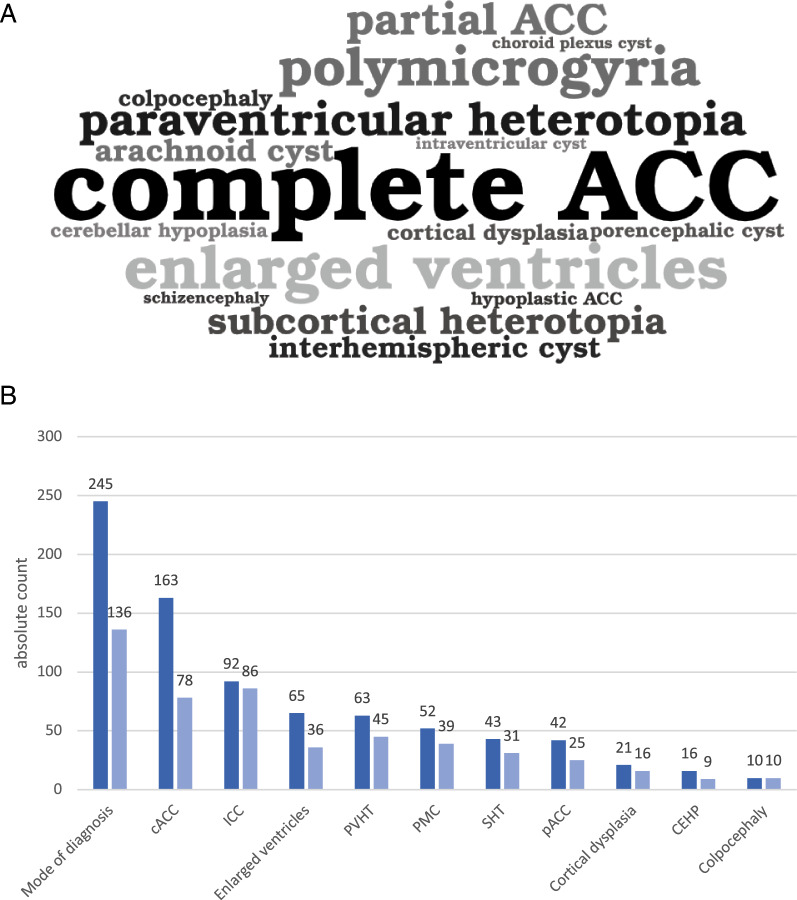
**a** Visualization of common neuroimaging findings in Aicardi syndrome as ‘wordle’. **b** Visualization of ten common neuroimaging findings in Aicardi syndrome as bar chart. Dark grey denominates findings in the entire cohort including cases from the pre-MRI era. Light grey indicates the findings when MRI was the mode of diagnosis. cACC, complete agenesis of corpus callosum; pACC, partial agenesis of corpus callosum; PMC, polymicrogyria; ICC, intracranial cyst; SHT, subcortical heterotopia; PVHT, periventricular nodular heterotopia; CEHP, cerebellar hypoplasia

Ophthalmological data was available for 242 children. CRL were present in 240 (99%) of all individuals. They were found bilaterally in 130 (54%) or only in the right or left eye in 17 (7%) and 22 (9%) cases respectively. In 71 (29%) cases the location was not stated. Optic nerve coloboma were reported in 75 (31%) cases. They were bilateral or restricted to the right or left eye in 26 (11%), 28 (12%) and 13 (5%) cases respectively. Microphthalmia was seen in 57 (24%) cases and most often in the right eye (27, 11%). For a detailed description of neuroradiological and ophthalmological findings see Fig. 4 and Supp. Tables 3 and 4.

#### Treatment data

In 103 of 245 (42%) children, details on seizure control were available. Seizure control was described as good/sufficient in 33 (32%, N = 103), as moderate/not satisfying in 28 (27%, N = 103) and as bad/intractable in 42 (41%, N = 103) cases. A seizure free interval was reported in 25 (10%) cases, in 10 (4%) cases lasting longer than 12 months. 3 (1%) patients could be weaned off medication at any given point in time. Seizure freedom at time of reporting was achieved in 15 (6%) cases. In 68 cases details on antiepileptic medication (AED) were given. The mean number of different AED at last follow-up was 1.9 (SD 1.0). The mean number of tried AED was 4.2 (SD 3.5). The maximum number of tried AED reported in one case was 16 at 19 years old. Other reported treatments included vagal nerve stimulation (VNS) in 6 cases, callosotomy in 2 cases and functional hemispherectomy and lobectomy in one case each.

### Predictors of clinical outcome

We explored possible predictors of clinical outcome by using Chi-squared analysis. Severe intellectual disability (${\chi }^{2}$=13.19, df = 4, *p* = 0.01), a complete absence of communicative skills (${\chi }^{2}$=18.195, df = 5, *p* = 0.003), a severe and global developmental delay (${\chi }^{2}$=17.16; df = 4, *p* = 0.002) and a severe motor impairment with inability to move (${\chi }^{2}$=15.89; df = 3, *p* = 0.001) were associated with a higher mortality rate. We explored the impact of age at onset and sidedness of chorioretinal lacunae on survival by using unbiased recursive partitioning. Age at onset above 2.1 could be associated with an unfavorable outcome. ROC Analysis showed a sensitivity of 91.3% and specificity of 54% for the cut-off at 2.1 months (see eFigure 1). No specific imaging abnormality or localization of imaging abnormalities or chorioretinal lacunae were associated with differences in survival.

### Variables influencing treatment success

**Fig. 5:**
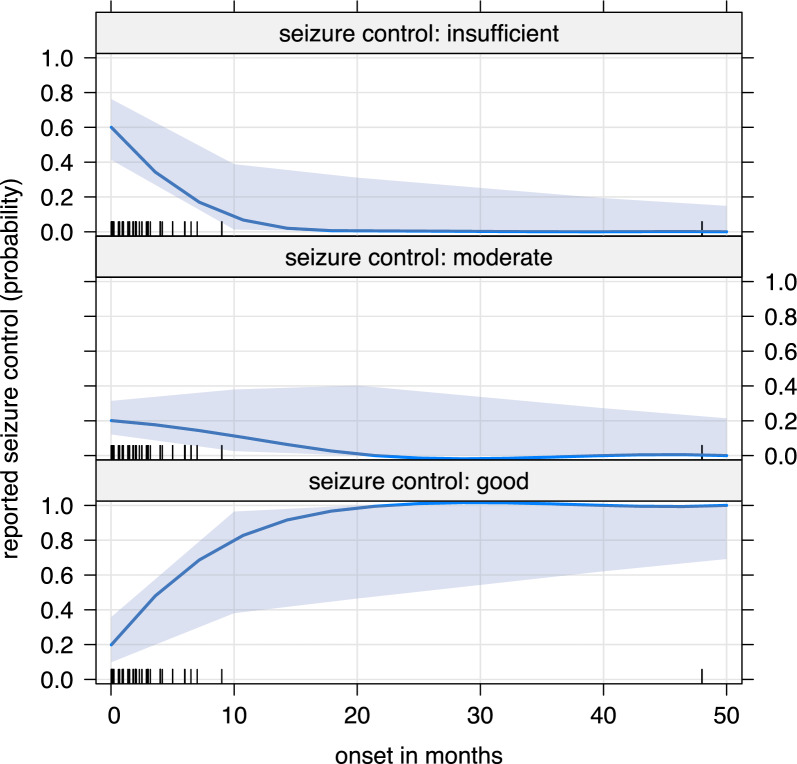
Degree of seizure control over age at first epileptic seizure. An early first seizure correlates with less favorable seizure control. (The lighter hue indicated the 95%)

The age at first epileptic seizure was associated with the seizure outcome. A later onset of epilepsy was associated with a better seizure control (Fig. 5). Seizure onset in the newborn period was associated with insufficient seizure control (${\chi }^{2}$=9.41, df = 1, *p* = 0.002). No association were found between specific neuroimaging features and seizure control or seizure freedom (Supp. Table 5 and 6).

## Discussion

We delineate the natural history of Aicardi syndrome by quantifying data from 245 individuals. Of those, 238 were girls and all reported individuals fulfilling the clinical criteria for AS genetically harbored two X-chromosomes, further arguing for a strong preference of females and contributing genetic factors for this syndromal developmental and epileptic encephalopathy. Aicardi syndrome can be diagnosed within a short delay after the occurrence of first clinical symptoms, which are usually epileptic seizures, mainly infantile spasms that are starting comparatively early around two months of life [23]. From our analysis, we can delineate Aicardi syndrome as a severe developmental and epileptic encephalopathy with an early onset of infantile spasms in most cases and with ID in nearly all (97%) affected girls. While 14% of affected individuals can have no or only mild ID, the majority developed moderate to severe ID. Communicative skills were equally impaired with only 17% of girls gaining the ability to speak words.

Our findings indicate a panethnic distribution pattern for AS (Fig. 1).

The age at onset from our large series is slightly earlier than in previously published cohorts and IS start earlier than in many other genetic DEEs with IS [11, 23, 24]. With increasing age, affected individuals are likely to develop several seizure types.

The prognosis of children with AS is reported to be generally poor with an estimated survival ranging from 40 to 62% during the first decade of life [4] to only 40% by age 15 [14]. The mean age at death in the whole cohort was 8.8 years (median 7.8 years), similar to findings from Glasmacher et al [12]. The mortality seems to decrease after birth and to steadily increase after the fourth year of life up to a peak at 16 years of age [4]. Given our large series, we here report an even higher percentage of deceased patients (15% vs. 11%) with a mean age at death of 3.92 years (median 1.75 years) compared to a previous large series of children [25]. The proportion of individuals reaching adulthood was comparable in our study with a probability of survival at the age of 20 years found in published reports of 0.62.

Mortality in AS is generally higher than in patients with isolated IS, where overall mortality is estimated at 5% by the age of 14 months [26]. While the mortality of AS around the first year of life is similar to other developmental and epileptic encephalopathies (DEEs) with IS, mortality rate at 5 years seems to be higher in children with AS (17% at the age of 5 years in our report vs. 8% at 4 years in unselected children with IS) [27]. Overall, our data suggests that mortality is increased in toddlers and smaller children.

Importantly and similar to other DEEs, earlier onset of epilepsy, especially in the neonatal period, was a predictor of a more severe course of disease (Fig. 3) [17, 24, 28–33]. Our findings also indicate that normal cognition is exceptional [14–17, 24, 34]. We provide most comprehensive data on MCDs, EEG and seizure semiologies in children with AS (see Suppl. Tables).

Cerebral asynchrony and hypsarrhythmia are described as consistent findings in EEG readings of AS patients. Hypsarrhythmia may not be present at diagnosis but can develop during the first months of life [24]. Both EEG markers are present in less than half of the cases at time of first symptoms.

In a survey of 69 children with AS, Glasmacher et al. previously reported on neuroimaging features. Our data shows that complete ACC in two thirds of cases or incomplete ACC in further 17% of cases are the hallmark of neuroimaging, findings that are mainly consistent to previous reports, importantly hypoplastic corpus callosum is a rarity in less than 4% of cases and should lead to consider differential diagnoses [12]. In this entire large series here MCDs were less frequent than in smaller previous studies focusing on brain imaging in AS [35, 36], most probably stemming from the lack of detailed imaging methods in some older reports. In cases with MRI or autopsy reports available, MCDs are present in the majority of cases (see eTable 3b and 3c) in accordance to newer reports [35]. In contrast to the findings of Masnada et al., burden of MCDs was not associated with seizure outcome in our study, suggesting that epilepsy in AS might also be related to neuronal changes other than just macroscopic cortical dysgenesis.

CRL were omnipresent in nearly all children screened for this study and they are seen in both eyes in about half of children, being the diagnostic hallmark and a specific finding in AS.

Treatment of seizures in AS is most often unsatisfactory and most affected girls had drug resistant epilepsy [17]. No superior drug or surgical procedure could be identified from the data curated here and we cannot support previous small series favoring specific drugs, such as vigabatrin [37]. Few descriptions of epilepsy surgery in AS are published and outcome is variable, ranging from seizure freedom to worsening of the seizures.

### Advantages and limitations of the study

The definition of the natural history of ultrarare conditions through systematic analysis of published natural history cases, has been carried out successfully in other ultrarare conditions [18, 38–42], where comprehensive prospective natural history studies are either unfeasible or will take many years to complete in a worldwide multicenter setting. There are several important limitations inherent to this method that have to be considered when interpreting our data. The estimation of treatment success is relied largely on statements made by the authors of the original case reports and studies, with likely different meanings for what is regarded as ‘successful’ therapy. Objectively stated data on seizure frequency was scarce in the reports and have been considered when possible. Information on intellectual functioning was most often stated in semi-quantitative terms or only in isolated aspects of daily functioning. Reported severity of disease is also likely affected by the revision of the diagnostic criteria and the changing imaging modalities. There might also be a publication bias towards more severe cases and more recently very mildly affected girls. Both might affect estimations given here.

As we defined requirements for inclusion into our analysis, valuable clinical information from non-included individuals might be missing. However, the need for stringent diagnostic criteria is regarded is most important to delineate specific developmental trajectories. For example, we excluded one report of a girl with retinoblastoma [43] because no clinical information on agenesis of corpus callosum or epilepsy was given. In line with this, published reports of neoplasms in AS might underly publication bias with a false increase of the prevalence of neoplasms.

Missing data may be an issue for quantitative retrospective natural history modeling as outlined previously [41. We therefore focused on hard endpoint variables such as survival, that was available for above 90%, whereas the lower data availability for the explored softer variables needs consideration when interpreting our study results. We took much effort to exclude data from multiply published cases wherever possible.

Despite these limitations, our study proofs helpful to define important outcome measures that will help to counsel parents and to inform future clinical studies in Aicardi syndrome.

### Conclusions

In this report, we provided quantitative natural history data including geographical localization of 245 published patients with Aicardi syndrome. We defined hard clinical endpoints such as the estimated survival. In addition, we explored associations between phenotypic details and presumable outcome. This data may help for counselling of families and for the planning of future clinical trials as one core element of quantitative systems pharmacology.

## Supplementary information

Supplementary Material 1.

Supplementary Material 2.

Supplementary Material 3.

Supplementary Material 4.

Supplementary Material 5.

Supplementary Material 6.

Supplementary Material 7.

Supplementary Material 8.

Supplementary Material 9.

Supplementary Material 10.

## Data Availability

All data generated or analyzed during this study are included in this published article. Additional raw data can be shared with researchers upon request.
