# Supplementary material for: Systematic quantitative modeling of the natural history of Aicardi syndrome: A cross sectional study of 245 published cases
Source: Orphanet J Rare Dis. 2024 Dec 4;19:457. doi: 10.1186/s13023-024-03375-8 (PMC11616230; doi:10.1186/s13023-024-03375-8)
Supplement: Supplementary file 1 — Supplementary Material 1. [file 13023_2024_3375_MOESM1_ESM.docx]

Supplementary Figure 1: ROC Analysis of variables “deceased” and “age at onset in months.” The cut-off 2.1 months reaches a sensitivity of 91% and a specificity of 54%
