# Supplementary material for: Systematic quantitative modeling of the natural history of Aicardi syndrome: A cross sectional study of 245 published cases
Source: Orphanet J Rare Dis. 2024 Dec 4;19:457. doi: 10.1186/s13023-024-03375-8 (PMC11616230; doi:10.1186/s13023-024-03375-8)
Supplement: Supplementary file 2 — Supplementary Material 2. [file 13023_2024_3375_MOESM2_ESM.docx]

Supplementary Table 1: Diagnostic criteria in original and revised form.

| Classic triad | Major features |
| --- | --- |
| ACC  CRL  Infantile spasms | MCD (most often polymicrogyria and heterotopias)  Intracerebral cysts (around 3^rd^ ventricle or choroid plexus)  Optic disc/nerve colobomas |

Two classic features plus at least two major features are strongly suggestive of AS. ACC = agenesis of corpus callosum. CRL = chorioretinal lacunae. MCD = malformation of cortical development.
