# Supplementary material for: Systematic quantitative modeling of the natural history of Aicardi syndrome: A cross sectional study of 245 published cases
Source: Orphanet J Rare Dis. 2024 Dec 4;19:457. doi: 10.1186/s13023-024-03375-8 (PMC11616230; doi:10.1186/s13023-024-03375-8)
Supplement: Supplementary file 3 — Supplementary Material 3. [file 13023_2024_3375_MOESM3_ESM.docx]

Supplementary Table 2: Dominant and non-dominant seizure types at time of diagnosis and at last follow-up.

| Dominant semiology  Non-dominant semiology | At time of diagnosis  (N = 215)  (N = 49) | At last follow-up  (N = 155)  (N = 35) |
| --- | --- | --- |
| Epileptic spasms | 175 (81.4%)  15 (30.6%) | 112 (72,2%)  5 (14,3%) |
| generalized tonic-clonic | 7 (3.3%)  11 (22.4%) | 8 (5,2%)  6 (17,1%) |
| Clonic | 2 (0.9%)  5 (10.2%) | 2 (1,3%)  3 (8,6%) |
| Tonic | 3 (1.4%)  10 (20.4%) | 9 (5,8%)  3 (8,6%) |
| Atonic | 0  4 (8.2%) | 1 (0,6%)  1 (2,9%) |
| Generalized nonmotor | 2 (0.9%)  5 (10.2%) | 1 (0,6%)  3 (8,6%) |
| Myoclonic | 4 (1.9%)  7 (14.3%) | 5 (3,2%)  9 (25,7%) |
| Focal-onset motor with impaired awareness | 3 (1.4%)  1 (2.0%) | 6 (3,9%)  2 (5,7%) |
| Focal-onset motor with retained awareness | 18 (8.4%)  16 (32.7%) | 11 (7,1%)  11 (31,4%) |
| Others | 2 (0.9%)  2 (4.1%) | 1 (0,6%)  3 (8,6%) |

Note: when possible, with adapted seizure semiologies to the classification of seizure semiology by the ILAE (International League against Epilepsy) from 2017. Given the context to Aicardi syndrome and its historic description, the term ‘infantile spasms’ was used throughout this study instead of ‘epileptic spasms’.
