# Supplementary material for: Systematic quantitative modeling of the natural history of Aicardi syndrome: A cross sectional study of 245 published cases
Source: Orphanet J Rare Dis. 2024 Dec 4;19:457. doi: 10.1186/s13023-024-03375-8 (PMC11616230; doi:10.1186/s13023-024-03375-8)
Supplement: Supplementary file 4 — Supplementary Material 4. [file 13023_2024_3375_MOESM4_ESM.docx]

Supplementary Table 3a: Overview of neuroradiological findings in all cases.

| Agenesis of corpus callosum |  | 240 (98.0%) |
| --- | --- | --- |
|  | Complete | 163 (66.5%) |
|  | Partial | 42 (17.1%) |
|  | Hypoplastic | 9 (3.7%) |
|  | Unspecified | 26 |
| Polymicrogyria |  | 52 (21.2%) |
|  | Bilateral | 30 (12.2%) |
|  | Right hemisphere | 4 (1.6%) |
|  | Left hemisphere | 5 (2.0%) |
|  | Unspecified | 13 |
| Schizencephaly |  | 5 (2.0%) |
| Intracranial cysts |  | 92 (37.6%) |
|  | Interhemispheric cyst | 39 (16.0%) |
|  | Arachnoid cyst | 21 (8.6%) |
|  | Porencephalic cyst | 11 (4.5%) |
|  | Choroid plexus cyst | 9 (3.7%) |
|  | Intraventricular cyst | 6 (2.4%) |
|  | Cerebellar cyst | 3 (1.2%) |
|  | Pineal gland cyst | 3 (1.2%) |
| Subcortical Heterotopia |  | 43 (17.6%) |
| Periventricular nodular heterotopia |  | 63 (25.7%) |
| Cortical dysplasia |  | 21 (8.6%) |
| Enlarged ventricles |  | 65 (26.5%) |
| Cerebellar hypoplasia |  | 16 (6.5%) |
| Delayed myelination |  | 5 (2.0%) |
| Colpocephaly |  | 10 (4.1%) |
| Arnold-Chiari malformation |  | 2 (0.8%) |
| Hydrocephalus |  | 1 (0.4%) |
| Lissencephaly |  | 1 (0.4%) |
| Hypoplastic pons |  | 1 (0.4%) |
