# Supplementary material for: Systematic quantitative modeling of the natural history of Aicardi syndrome: A cross sectional study of 245 published cases
Source: Orphanet J Rare Dis. 2024 Dec 4;19:457. doi: 10.1186/s13023-024-03375-8 (PMC11616230; doi:10.1186/s13023-024-03375-8)
Supplement: Supplementary file 5 — Supplementary Material 5. [file 13023_2024_3375_MOESM5_ESM.docx]

Supplementary Table 3b: Overview of neuroradiological findings in all cases with MRI reported (N=136)

| Agenesis of corpus callosum |  | 113 (83.1%) |
| --- | --- | --- |
|  | Complete | 78 (57.4%) |
|  | Partial | 25 (18.4%) |
|  | Hypoplastic | 7 (5.1%) |
|  | Unspecified | 3 (2.2%) |
| Polymicrogyria |  | 39 (28.7%) |
|  | Bilateral | 23 (16.9%) |
|  | Right hemisphere | 4 (2.9%) |
|  | Left hemisphere | 5 (3.7%) |
|  | Unspecified | 0 |
| Schizencephaly |  | 5 (3.7%) |
| Intracranial cysts |  | 86 (63.2%) |
|  | Interhemispheric cyst | 30 (22.1%) |
|  | Arachnoid cyst | 14 (10.3%) |
|  | Porencephalic cyst | 1 (0.7%) |
|  | Choroid plexus cyst | 6 (4.4%) |
|  | Intraventricular cyst | 4 (2.9%) |
| Subcortical Heterotopia |  | 31 (22.8%) |
| Periventricular nodular heterotopia |  | 45 (33.1%) |
| Cortical dysplasia |  | 16 (11.8%) |
| Enlarged ventricles |  | 36 (26.5%) |
| Cerebellar hypoplasia |  | 9 (6.6%) |
| Colpocephaly |  | 10 (7.4%) |
