# Supplementary material for: Systematic quantitative modeling of the natural history of Aicardi syndrome: A cross sectional study of 245 published cases
Source: Orphanet J Rare Dis. 2024 Dec 4;19:457. doi: 10.1186/s13023-024-03375-8 (PMC11616230; doi:10.1186/s13023-024-03375-8)
Supplement: Supplementary file 6 — Supplementary Material 6. [file 13023_2024_3375_MOESM6_ESM.docx]

Supplementary Table 3c: Neuroradiological findings in autopsy cases (N=12)

| Agenesis of corpus callosum |  |  | 12 (100%) |
| --- | --- | --- | --- |
|  | Complete |  | 8 (66.7%) |
|  | Partial |  | 4 (33.3%) |
| Polymicrogyria |  |  | 11 (91.7%) |
|  | Bilateral |  | 6 |
|  |  | Right frontal | 4 |
|  |  | Right parietal | 2 |
|  |  | Right temporal | 0 |
|  |  | Right occipital | 0 |
|  |  | Left frontal | 2 |
|  |  | Left parietal | 2 |
|  |  | Left temporal | 0 |
|  |  | Left occipital | 0 |
|  |  | Left cingulate gyrus | 1 |
|  | Unspecified |  | 5 |
| Subcortical Heterotopia |  |  | 5 (41.7%) |
| Periventricular nodular heterotopia |  |  | 7 (58.3%) |
| Cerebellar hypoplasia |  |  | 3 (25.0%) |
| Abnormal basal ganglia |  |  | 1 (8.3%) |
| Hippocampal malrotation |  |  | 1 (8.3%) |
