# Supplementary material for: Systematic quantitative modeling of the natural history of Aicardi syndrome: A cross sectional study of 245 published cases
Source: Orphanet J Rare Dis. 2024 Dec 4;19:457. doi: 10.1186/s13023-024-03375-8 (PMC11616230; doi:10.1186/s13023-024-03375-8)
Supplement: Supplementary file 7 — Supplementary Material 7. [file 13023_2024_3375_MOESM7_ESM.docx]

Supplementary Table 4: Overview of ophthalmological findings in all cases.

|  | Absolute number (b/r/l/u) | Percentage in % (N=242) |
| --- | --- | --- |
| Chorioretinal lacunae | 130 / 17 / 22 / 71 | 53.7 / 7.0 / 9.1 / 29.3 |
| Optic nerve coloboma | 26 / 28 / 13 / 8 | 10.7 / 11.6 / 5.4 / 3.3 |
| Iris coloboma | 0 / 4 / 2 / 0 | - / 1.7 / 0.8 / - |
| Retinochoroidal coloboma | 0 / 0 / 1 / 0 | - / - / 0.4 / - |
| Retroocular coloboma/cyst | 0 / 4 / 3 / 2 | - / 1.7 / 1.2 / 0.8 |
| Morning glory papilla | 1 / 2 / 1 / 0 | 0.4 / 0.8 / 0.4 / - |
| Microphthalmia | 9 / 27 / 17 / 4 | 3.7 / 11.2 / 7.0 / 1.7 |
| Persistent fetal vasculature | 0 / 3 / 2 / 0 | - / 1.2 / 0.8 / - |
| Optic hypoplasia | 12 / 10 / 10 / 7 | 5.0 / 4.1 / 4.1 / 2.9 |
| Optic aplasia | 1 / 1 / 0 / 0 | 0.4 / 0.4 / 0 / 0 |
| Atrophic papilla | 0 / 1 / 0 / 0 | - / 0.4 / - / - |
| Dysplastic disc | 0 / 2 / 1 / 0 | - / 0.8 / 0.4 / - |
| Iris cyst | 0 / 0 / 1 / 0 | - / - / 0.4 / - |
| Iridic synechia | 0 / 0 / 1 / 0 | - / - / 0.4 / - |
| Posterior synechia | 0 / 2 / 0 / 0 | - / 0.8 / - / - |
| Retinal detachment | 2 / 4 / 6 / 0 | 0.8 / 1.7 / 2.5 / - |
| Cataract | 0 / 2 / 0 / 0 | - / 0.8 / - / - |
| Nystagmus | 0 / 0 / 1 / 3 | - / - / 0.4 / 1.2 |
| Ptosis | 2 / 2 / 2 / 0 | 0.8 / 0.8 / 0.8 / - |
| Proptosis | 0 / 2 / 2 / 0 | - / 0.8 / 0.8 / - |
| Horner syndrome | 0 / 1 / 0 / 0 | - / 0.4 / - / - |
| Strabism | 14 | 5.8 |
| Aniridia | 2 | 0.8% |
| retinopathy of prematurity | 3 | 1.2 |

Reports on ophthalmological findings were available in 242 cases. Stated as b/r/l/u, where b=bilateral, r=right eye, l=left eye, u=unspecified.
