# Supplementary material for: Systematic quantitative modeling of the natural history of Aicardi syndrome: A cross sectional study of 245 published cases
Source: Orphanet J Rare Dis. 2024 Dec 4;19:457. doi: 10.1186/s13023-024-03375-8 (PMC11616230; doi:10.1186/s13023-024-03375-8)
Supplement: Supplementary file 8 — Supplementary Material 8. [file 13023_2024_3375_MOESM8_ESM.docx]

Supplementary Table 5: Results of Pearson’s Chi-squared tests for the effect of neuroimaging features on seizure control

|  | X-squared | p-value |
| --- | --- | --- |
| Agenesis of corpus callosum | 0.80238 | 0.6695 |
| Interhemispheric cyst | 1.8553 | 0.3955 |
| Arachnoidal cyst | 1.316 | 0.5179 |
| Choroid plexus cyst | 0.17329 | 0.917 |
| Ventricular cyst | 1.8015 | 0.4063 |
| Porencephalic cyst | 1.1454 | 0.564 |
| Pineal gland cyst | 0.13691 | 0.9338 |
| Cerebellar cyst | 1.6208 | 0.4447 |
| Polymicrogyria | 0.10546 | 0.9486 |
| Schizencephaly | 0.16088 | 0.9227 |
| Cortical dysplasia | 2.7186 | 0.2568 |
| Subcortical heterotopia | 0.74669 | 0.6884 |
| Subependymal heterotopia | 1.4161 | 0.4926 |
| Cerebellar hypoplasia | 0.68434 | 0.7102 |
| Cerebellar dysplasia | 1.7025 | 0.4269 |
| Dandy walker malformation | 1.5056 | 0.4711 |
| Delayed myelination | 1.4601 | 0.4819 |
| Enlarged ventricles | 2.1792 | 0.3363 |
| Enlarged cisterna magna | 1.5048 | 0.4712 |
| Colpencephaly | 1.2634 | 0.5317 |
| Pons hypoplasia | 1.5873 | 0.4522 |
| Hydrocephalus | 2.0328 | 0.3619 |

df=2 for all calculations. No neuroimaging feature is significantly correlated with seizure control.
