# Supplementary material for: Systematic quantitative modeling of the natural history of Aicardi syndrome: A cross sectional study of 245 published cases
Source: Orphanet J Rare Dis. 2024 Dec 4;19:457. doi: 10.1186/s13023-024-03375-8 (PMC11616230; doi:10.1186/s13023-024-03375-8)
Supplement: Supplementary file 9 — Supplementary Material 9. [file 13023_2024_3375_MOESM9_ESM.docx]

Supplementary Table 6: Pearson’s Chi-squared tests with Yates’ continuity correction for the effect of neuroimaging features on seizure freedom at time of reporting.

|  | X-squared | p-value |
| --- | --- | --- |
| Agenesis of corpus callosum | 4.39e-31 | 1 |
| Interhemispheric cyst | 0.0666 | 0.797 |
| Arachnoidal cyst | 8.578e-31 | 1 |
| Choroid plexus cyst | 2.414e-31 | 1 |
| Ventricular cyst | 2.923e-31 | 1 |
| Porencephalic cyst | 0.121 | 0.728 |
| Pineal gland cyst | 2.923e-31 | 1 |
| Cerebellar cyst | 1.157 | 0.282 |
| Polymicrogyria | 0 | 1 |
| Schizencephaly | 1.370 | 1 |
| Cortical dysplasia | 7.479e-31 | 1 |
| Subcortical heterotopia | 0.256 | 0.613 |
| Subependymal heterotopia | 0.052 | 0.820 |
| Cerebellar hypoplasia | 2.566e-31 | 1 |
| Cerebellar dysplasia | 3.034e-29 | 1 |
| Dandy walker malformation | 0.004 | 0.948 |
| Delayed myelination | 0.226 | 0.635 |
| Enlarged ventricles | 0.052 | 0.819 |
| Enlarged cisterna magna | 1.417e-30 | 1 |
| Colpencephaly | 1.680e-30 | 1 |
| Pons hypoplasia | 1.140 | 0.286 |
| Hydrocephalus | 0.027 | 0.869 |

df=1 for all calculations. No neuroimaging feature is significantly correlated with seizure freedom at time of reporting.
