# Supplementary material for: Systematic quantitative modeling of the natural history of Aicardi syndrome: A cross sectional study of 245 published cases
Source: Orphanet J Rare Dis. 2024 Dec 4;19:457. doi: 10.1186/s13023-024-03375-8 (PMC11616230; doi:10.1186/s13023-024-03375-8)
Supplement: Supplementary file 10 — Supplementary Material 10. [file 13023_2024_3375_MOESM10_ESM.docx]

Supplementary Table 7: Overall survival

| time | n_risk | n_event | survival | std_error | Lower 95% CI | Upper 95% CI |
| --- | --- | --- | --- | --- | --- | --- |
| 1 | 165 | 12 | 0.941 | 0.0165 | 0.909 | 0.974 |
| 2 | 133 | 9 | 0.881 | 0.0247 | 0.834 | 0.931 |
| 3 | 115 | 2 | 0.867 | 0.0264 | 0.816 | 0.920 |
| 4 | 92 | 2 | 0.849 | 0.0288 | 0.794 | 0.907 |
| 5 | 81 | 1 | 0.838 | 0.0303 | 0.781 | 0.900 |
| 6 | 69 | 0 | 0.838 | 0.0303 | 0.781 | 0.900 |
| 7 | 62 | 1 | 0.825 | 0.0327 | 0.763 | 0.891 |
| 8 | 56 | 0 | 0.825 | 0.0327 | 0.763 | 0.891 |
| 9 | 46 | 1 | 0.808 | 0.0361 | 0.740 | 0.882 |
| 10 | 39 | 0 | 0.808 | 0.0361 | 0.740 | 0.882 |
| 11 | 34 | 0 | 0.808 | 0.0361 | 0.740 | 0.882 |
| 12 | 33 | 1 | 0.783 | 0.0425 | 0.704 | 0.871 |
| 13 | 26 | 1 | 0.753 | 0.0504 | 0.660 | 0.859 |
| 14 | 23 | 1 | 0.720 | 0.0579 | 0.615 | 0.843 |
| 15 | 19 | 0 | 0.720 | 0.0579 | 0.615 | 0.843 |
| 16 | 17 | 0 | 0.720 | 0.0579 | 0.615 | 0.843 |
| 17 | 16 | 1 | 0.675 | 0.0696 | 0.552 | 0.827 |
| 18 | 15 | 0 | 0.675 | 0.0696 | 0.552 | 0.827 |
| 19 | 15 | 0 | 0.675 | 0.0696 | 0.552 | 0.827 |
| 20 | 12 | 1 | 0.619 | 0.0835 | 0.475 | 0.806 |
|  |  |  |  |  |  |  |
